# Design of a sensor network for the quantitative analysis of sport climbing

**DOI:** 10.3389/fspor.2023.1114539

**Published:** 2023-02-20

**Authors:** Alessandro Colombo, Ramon Maj, Marita Canina, Francesca Fedeli, Nicolò Dozio, Francesco Ferrise

**Affiliations:** ^1^ Department of Electronics, Information, and Bioengineering, Politecnico di Milano, Milano, Italy; ^2^ Department of Design, Politecnico di Milano, Milano, Italy; ^3^ Fightthestroke Foundation ETS, Milano, Italy; ^4^ Department of Mechanical Engineering, Politecnico di Milano, Milano, Italy

**Keywords:** sport climbing, sensors, motion analysis, performance, instrumented climbing hold

## Abstract

We describe the design of a modular sensorized climbing wall for motion analysis in a naturalistic environment. The wall is equipped with force sensors to measure interaction forces between the athlete and the wall, which can be used by experienced instructors, athletes, or therapists, to gain insights into the quality of motion. A specifically designed triaxial load cell is integrated into each hold placement, invisible to the climber, and compatible with standard climbing holds. Data collected through the sensors is sent to an app running on a portable device. The wall can be adapted to different uses. To validate our design, we recorded a repeated climbing activity of eleven climbers with varying degrees of expertise. Analysis of the interaction forces during the exercise demonstrates that the sensor network design can provide valuable information to track and analyze exercise performance changes over time. Here we report the design process as well as the validation and testing of the sensorized climbing wall.

## Introduction

Sport is the quintessential domain of body feeling and emotional thinking, diametrically opposite to the abstract thinking of mathematics. Despite this, quantitative thinking has long been leaking into sports, starting from early attempts to rationalize and statistically analyze performances in major sporting events: think of the famous Moneyball events of the early ’90, which have their roots in E. Cook’s work (1). Sports analytics is now becoming a global trend, fueled by success stories in most major sports as well as by the availability of miniaturized and wearable sensors, themselves a byproduct of Moore’s law and the popularization of MEMS devices in smartphones (2). It feeds a growing market of tracking and measuring devices with increasingly high performance and lower costs that allow athletes or practitioners to monitor performance, analyze improvements, and gain motivation. These technologies can be wearable (3, 4) or integrated into the sporting equipment (5, 6), depending on the domain and measuring requirements.

In this sporting data deluge, sport climbing has so far remained a relatively dry land. This is mainly due to two factors: sport climbing has only recently started to become a major trend (it entered the Olympic Games in Tokyo 2021), and it has been so far a low-budget activity. Sports scientists have nonetheless attempted to introduce in climbing the likes of the quantitative analysis that are now common in other sports. The physiology of climbing has been investigated through experimental testing in high-level athletes since the early ’90s (see (7) for a review of the early works). Quantitative analysis of climbing movement and equilibrium was initially addressed in (8,9), and later developed in (10–13). Finger strength, posture, and their effect on climbing were investigated in (14–18). Finally, force sensors were used to measure contact forces between athlete and climbing wall (19–26), while body pose reconstruction from a video stream was used in (27) to study climbing movement.

In most of the above studies that involve force measurements, data was collected using commercial, general-purpose force sensors adapted to fit the task (with the notable exception of (21,23)). Furthermore, the sensor networks were designed for use in a laboratory environment, meaning that in many cases the sensors were visible to the climber and potentially altered the climbing experience. The aim of these studies was to use or test the viability of a laboratory measurement setup. As sport climbing is transitioning from a niche activity to a mainstream sport, there is growing interest in the design of a sensorized climbing wall with scalable sensing technology (i.e., reasonable cost and low complexity of assembly of a large sensor network), and a user interface designed for coaches and athletes rather than engineers. Means to acquire and analyze climbing motion may also benefit athletes by way of improving the understanding of extremities workloads and overuse. Shoulders and fingers are known to be the most common injury site, and overuse is the most common cause (28–30).

On this basis, through a set of co-design sessions (31–34) involving climbers, climbing instructors, and physiotherapists, we identified the requirements and we realized the first prototype of a scalable sensorised wall that is suitable for usage out of the university labs. In this paper, we report the outcomes of the co-design sessions, and the details of the resulting design, including the structure of the acquisition hardware and software that we developed. We then report preliminary data obtained from testing a prototype of the sensorized wall with 11 participants in a simple climbing drill.

## Methods

### Analysis of the requirements

To guide the design of the sensorized wall we conducted eight in-depth, semi-structured expert interviews (35). Six experts were identified from the climbing field: two climbers, two climbing instructors, and two physiotherapists. Two more experts were selected from a research institute (engineers/climbers). They were asked to share their personal context-specific knowledge instead of providing official association statements (36). The qualitative approach with semi-structured interviews provides open-ended answers, not quantitative data. These interviews, and the needs that we identified, were turned into design requirements (see Table 1) through a further round of literature review and interaction between the research team and the experts.

**Table 1:** An overview of the main requirements identified through the interviews.

Requirements
(R1) 3-DOF force sensors, integrated into the wall, invisible from the climber side, with M10 attachment compatible with standard climbing holds.
(R2) Sensor able to resolve 5N.
(R3) Force measurement range up to 1600N independently of point of application.
(R4) Recorded force signals should be synchronized, to allow composition and comparison of signals between holds
(R5) The measured signals should be visualizable during or immediately after the exercise on a portable device, expressed in kgf, and should be stored for subsequent analysis.
(R6) The graphical interface should refer recorded signals to a positional representation of the holds on the wall and should adapt to changes in holds and sensors’ positions.

One of the common needs highlighted by the experts was to obtain a quantitative measure related to weight distribution, and more in general a measure directly or indirectly related to body pose, and athletic and motor performance during regular climbing tasks in a variety of climbing scenarios, with a variety of hold shapes and arrangements. This was however combined with the need to avoid wearable devices and modifications to the surface of the wall that could interfere with athletic gestures. To meet this requirement, the main available options are camera-based motion capture or force sensors embedded in the climbing holds. The former could provide a direct measure of body pose, while the latter provide more direct information regarding the load distribution and may potentially convey information that is more difficult to gather by the climbing instructor through direct observation of the climbing exercise. For this reason we opted for force sensors, though the integration of camera-based motion capture was planned as a follow-up and is currently being pursued. A potentially interesting solution was to integrate force and grip pressure sensors in the holds. This, however, would have meant modifying the holds structure: we would no longer have been able to use standard climbing holds, and this would have greatly restricted the variety of scenarios that can be realized at a reasonable cost. Climbing instructors and physiotherapists in particular highlighted the importance of being able to use a wide variety of hold shapes and textures and to easily replace or rotate holds on a climbing route. We, therefore, chose to use force sensors integrated into the wall surface and designed to allow the mounting of standard climbing holds with standard M10 attachment screws. While (21) explored the opportunity of using 1 or 2-Degrees-Of-Freedom (DOF) force sensors to instrument a climbing wall, with the measurement axes lying in the plane parallel to the wall, a full 3-DOF sensor may provide more complete information regarding the pose of the climber’s body. We, therefore, decided to follow the route of a 3-DOF sensor, with a complete redesign of the sensor geometry to limit costs and manufacturing complexity.

Our need-finding session also highlighted how a wall might be used by novice and expert climbers, with an estimated body weight ranging from 20kg for younger kids to 80 kg, which is the reference weight of an adult climber in the EN norms on climbing equipment. Physiotherapist also highlighted the potential interest in using the wall during therapy for children with mild motor disabilities, in particular hemiplegia. For this, sensors would need to resolve the force generated by the child’s affected side. We estimated that a resolution of 5 N would allow resolving the force generated by the arm of a child passively posed on a hold. The measurement range thus should go from 5 N, to over 1600 N, assuming a factor 2 in a dynamic load of an 80 kg climber. Note that, like the 80 kg climber weight, the factor 2 was chosen in accordance with the parameters used to define the structural integrity requirements of climbing holds in EN 12572-3. Because of the need to use the sensors in several exercises and to simplify reconfiguration, the sensor design should also not need recalibration when holds are changed, providing measurements that are independent of the hold shape and size. Due to the variety of commercially available hold shapes, this led us to formulate a requirement that the sensing device should provide a measure of the force vector that is as much as possible insensitive to the point of application of the force on the hold. Finally, in order to be able to compose data from multiple sensors, the force signals should be synchronized and referred to a common reference frame.

Concerning software requirements, an important aspect that emerged from the needs expressed by all the experts was to provide real-time access to the collected data, together with the possibility to visualize it and perform subsequent statistical analysis. The measured signals should be, therefore, visualizable by the climber, climbing instructor, or physiotherapist, on a portable device (smartphone, tablet, laptop), through an interface that is simple enough to be used during or immediately after the exercise without technical training. Information gathered through the sensors should also be represented so as to be understandable by different users. As an example, the climbing instructors might be interested in storing and analyzing the numerical load information later. The climber might instead be interested in a graphical, simplified, and real-time representation of the same information. In both cases, referring signals to a graphical representation of the wall was considered to aid interpretation. We also found a preference for expressing forces in kgf as opposed to N, which allowed simpler considerations regarding body weight distribution. Finally, in order to comply with the need to reconfigure the wall easily, data visualization and sensors-related aspects of the application interface should easily adapt to a change in sensor position, the addition or removal of some sensors, or the repositioning of the holds on the wall. Table 1 summarizes the main requirements that emerged through this process.

### Hardware implementation

**Figure 1:**
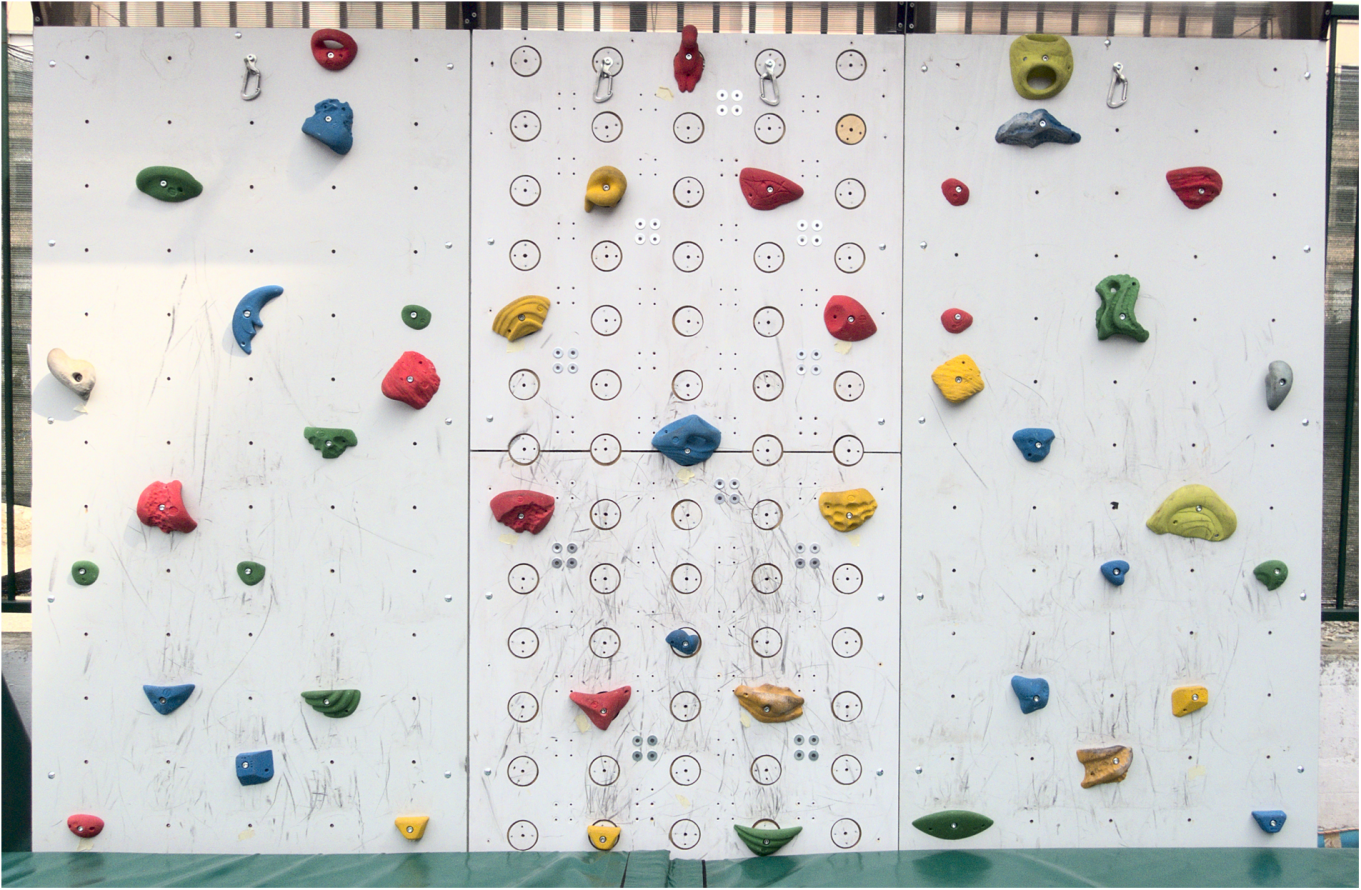
Prototype of the sensorized wall. The wall is composed of three modules side by side; in this case, only the central module is equipped with sensors.

At the time of implementing the design, we had the chance of installing the sensorized wall in a public sporting center that caters mostly to children aged 6 to 13yo. As a consequence, we decided to size the structure for these users. The structure is composed of independent modules (3 side-by-side, in Figure 1), each 1200 mm wide and 2500 mm high, supported by a steel frame that allows a variable inclination of the wall of about $15^{\circ }$ either side from the vertical. The wall could of course be realized with taller modules up to the maximum height of 4.5 m allowed by norm EN 12572-2 (37) with minimal changes to the design.

The steel frame, the 20 mm-thick plywood covering, and all structural details were designed according to EN 12572-2. This allowed us to open the wall to public use. The structure was designed for outdoor use, and a cover was added to protect the sensors from rain and to eliminate the possibility of young users climbing over the wall.

In the wall in Figure 1, the central module is sensorized, while the two side modules are not. Independently of whether a sensorized or non-sensorized panel is mounted, the distance between hold attachments is 230 mm horizontally and 180 mm vertically, and holds are connected to the wall (or the sensors) through standard M10 screws: the sensor was specifically designed to provide standard M10 attachment for commercial holds and to provide a sufficient frontal surface to fix holds up to 200 mm in diameter securely.

To minimize noise and allow easy reconfiguration of the sensors, we equipped each sensor with a dedicated data acquisition board, itself custom-designed and equipped with a 6-channel analog to digital converter with 24-bit resolution and a sampling frequency of 80 Hz (based on the AD7124-4 chip), and a CAN bus interface (MCP2515). All sensors, therefore, behave like independent nodes of a CAN network and can be mounted and removed without any need to modify the hardware or software configuration of the other sensors.

**Figure 2:**
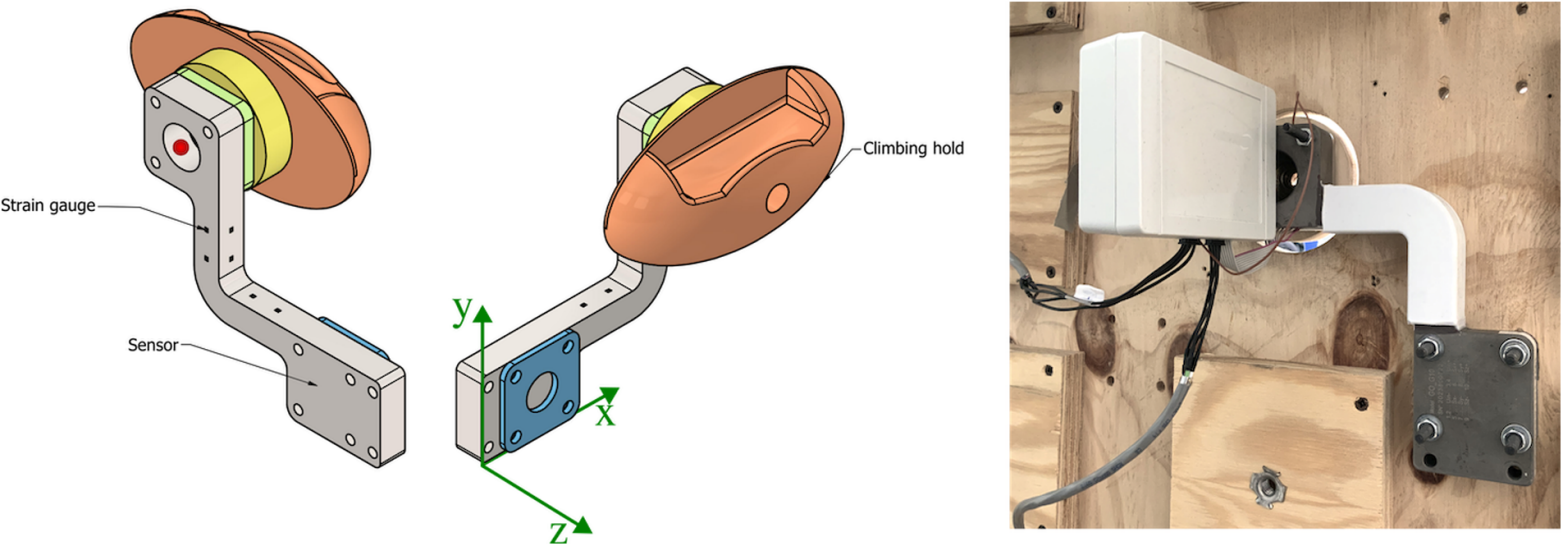
Sensor components, on the left, and a picture of a mounted sensor, taken from behind the wall. In the drawing, the sensing element is represented in gray and the strain gauges in black, the green and blue components are spacers, while the yellow component is a wooden disk. The green reference frame refers to the axes used in the sensor characterization, and the red point on the top left of the figure is the reference with respect to which testing offsets are measured, as described in the Experimental Design section. In the picture, the box on the left encases the acquisition board and is permanently attached to the sensing element.

To minimize the production cost of the 3-DOF sensor we redesigned the sensing element from scratch. The shape, obtained from 20 mm thick steel slabs, was designed to require a single cut on a 2-DOF machine while providing easy access to the surfaces that host the strain gauges, to simplify the process of applying the strain gauges and electronics. The resulting design, which is patent-pending, is reported in Figure 2. The shape of the sensor and the placement of the strain gauges were optimized through FEM analysis to provide good resolution over the range of ∼5 to 1600 N and to withstand loads of up to 2400 N without damage. This range was obtained by assuming the maximum dynamic load of 1600 N, multiplied by a 1.5 safety factor. While the sensor bends slightly under load, FEM analysis predicted that during normal use (i.e., under loads of up to 1600 N) the hold would move by less than 2 mm. The housing of the sensors in the frontal panel was therefore designed to allow for this displacement without contact between the sensing element and the wall. The wooden disk, 80 mm in diameter, protrudes 2 mm from the wall to avoid contact between the climbing hold and the wall under the expected load range provides the surface supporting the hold, and hosts a standard M10 fixing in the center. These disks are clearly visible in the central panel in the wall of Figure 1. Notice that, even though in principle high-level climbers could use the 2mm step as an additional grip, during our test this did not appear to be the case. Note that to mount or move a sensor the user must access the back of the wall. The mounting procedure is quite simple: the sensor is attached through 4 screws, each sensor is equipped with its own acquisition board, and power and data are brought to the acquisition board through a 4-wire, hot-swappable, daisy chain connection. Moving or adding a sensor thus requires attaching 4 screws and connecting the sensor as an arbitrary segment of the daisy chain. When it came to building the sensors, the total cost for the low-volume industrial production of a batch of sensors by a load-cell manufacturer, plus the cost to have the custom-made acquisition boards assembled by a PCB manufacturer was about 310C€ per sensor, in line with the low-cost (and 2-DOF) implementation suggested in (21). These features satisfy (R1)–(R3).

### Software implementation

The goal of the software interface is to provide a structured tool to quantitatively evaluate the performance of the climber in real-time and to easily record and export data for subsequent analysis with other software and to simplify the reconfiguration of the sensorized wall, according to (R4)–(R6).

From the point of view of the software interface, the climbing wall was set up as a server that provides data coming from the sensors, while the software interface itself is a mobile application running on a mobile device. The server implements the data collection from the sensors connected through the CAN bus. Data is then made available through a Representational State Transfer (REST) web service and a set of threads and queues. At any given time, a client can connect to the REST service and download the latest data coming from the sensors. The client side of the application is represented by a mobile application running on a modern tablet. In our setup, we used an Apple iPad in two different versions (Apple iPad Pro 10.2${}^{\prime \prime }$ 2016, and Apple iPad Pro 13${}^{\prime \prime }$ 2020), running iOS 15. The client app was however written in React Native to allow a simple porting to Android, and has been successfully tested on Android 12. We defined this component as a REST service on the mobile side since it was not possible to define a streaming API to retrieve data. For this reason, the mobile application regularly polls the REST web service to retrieve the data when the application is in recording mode, and the REST web service packs the available data in a JSON structure before returning it to the caller.

On the client side, the mobile application provides four main environments:

• a Setup environment used to configure the sensor network when sensors or holds placement is changed,
• a User Detail environment used to assign identification data to an athlete before recording,
• a Data Acquisition environment used to record data
• a Data Management and Recording Analysis environment used to visualize data and export it.

**Figure 3:**
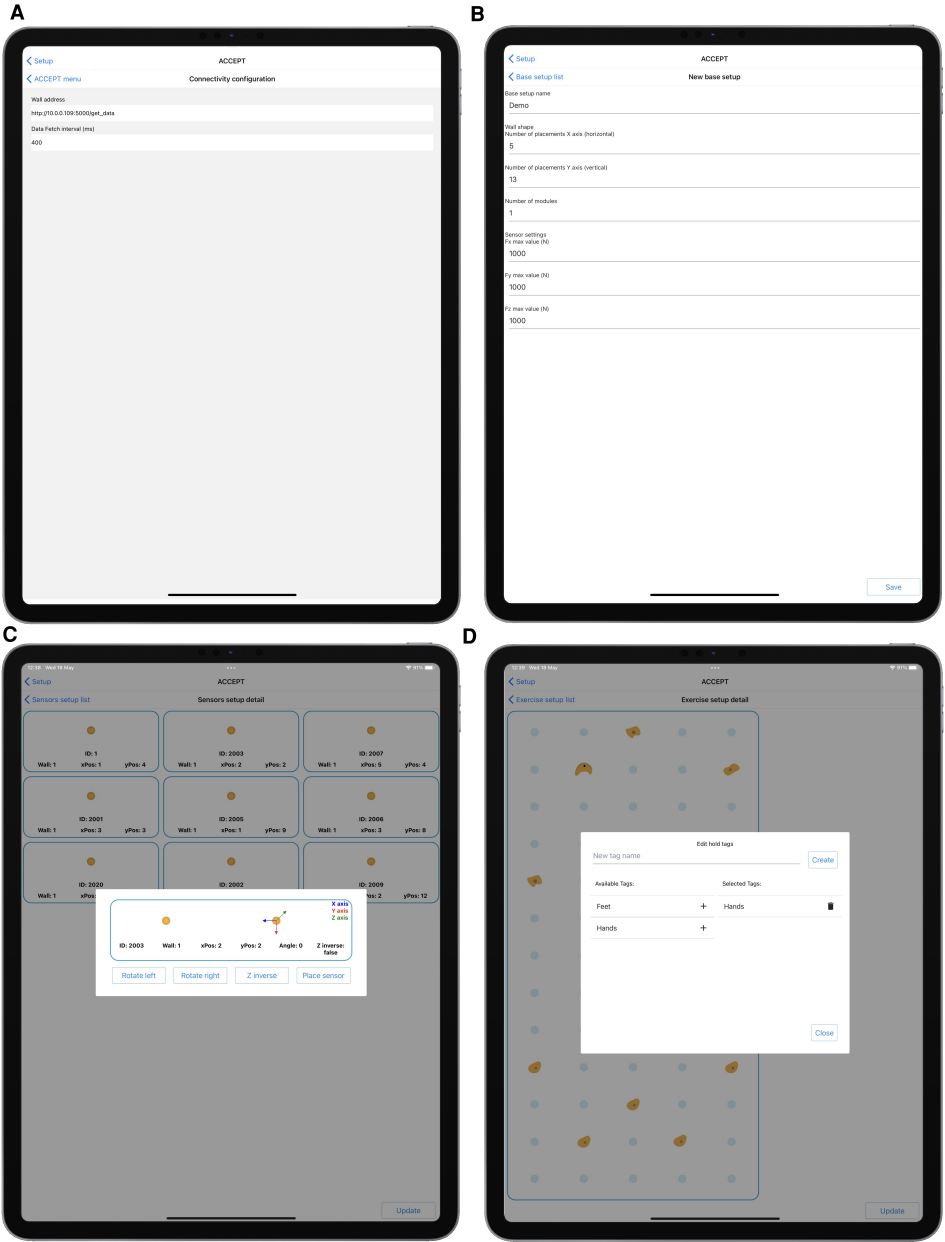
Setup environment. (**A**) wall address setup; (**B**) wall geometry setup; (**C**) association of sensors to placements on the wall grid; (**D**) definition of hold tags and selection of holds that compose a climbing problem. In panel (**C**), the position coordinates of the placements originate in the lower left corner, position (1,1) being the lower left placement; xPos is the horizontal position, yPos the vertical one. Once a hold is placed on the grid, the same environment displays a virtual representation of the wall and allows the user to assign a shape to the hold, chosen from a menu of pre-defined shapes. This step of the setup is not depicted in the figure, but the virtual representation that ensues is visible in panel (**D**).

The Setup environment, shown in Figure 3, allows to define the parameters needed to connect a tablet to the acquisition system (panel A), to define the number of possible hold placements on the sensorized wall, which are arranged on a regular grid (panel B), to associate sensors and holds with specific placements (panel C), and to define the group of holds that constitute a given climbing problem, attaching optional tags to specific holds (panel D). In particular, when associating sensors to placements, the Setup environment allows to rotate of the sensor reference frame (Figure 3C) so that the reference frames of all sensors are aligned, with the x axis parallel to the horizontal edge of the wall and pointing right, the y axis parallel to the vertical edge and pointing up, the z axis pointing out of the wall. This allows having a coherent framework for all sensors, which is necessary when composing forces measured on different holds. After having assigned a hold to a placement on the grid, the user can select a visual representation of the hold from a menu of pre-defined shapes (this menu and the corresponding configuration step are not displayed in the figure). This helps to more easily relate the wall virtual representation, visible in Figures 3 and 5, with the real wall, visible in Figure 7. Furthermore, as mentioned before, the Setup environment allows attaching one or more user-defined tags to the sensors (Figure 3D), to automate subsequent classification and analysis in the Data Management and Recording Analysis environment. In the tests that were performed for this study, since we had dedicated distinct sets of holds to hands and feet, we tagged as *Hands* the upper 5 holds and *Feet* the bottom five ones (see Figure 3, bottom right panel).

**Figure 4:**
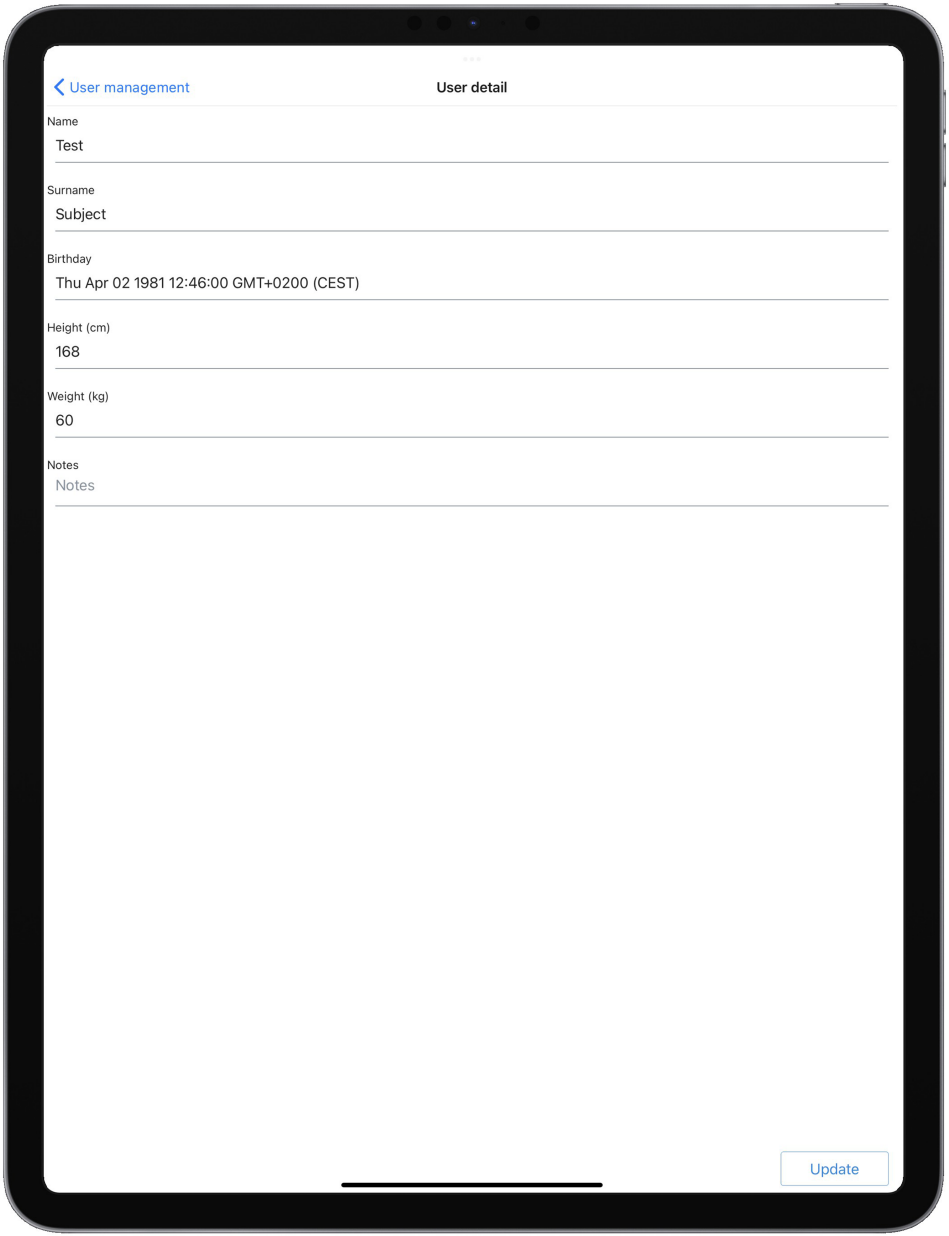
User Detail environment. User data defined in this environment can be attached to a recording.

The User Detail environment, in Figure 4, is used to insert user data that will be attached to a recording. In particular, we decided to include first and last names, age, height, and weight, the last two being relevant to parametrize many biomechanical models. The weight is also used in the Data Management and Recording Analysis environment (Figure 6B), to visually compare the total force expressed on tagged holds with the climber’s body weight.

**Figure 5:**
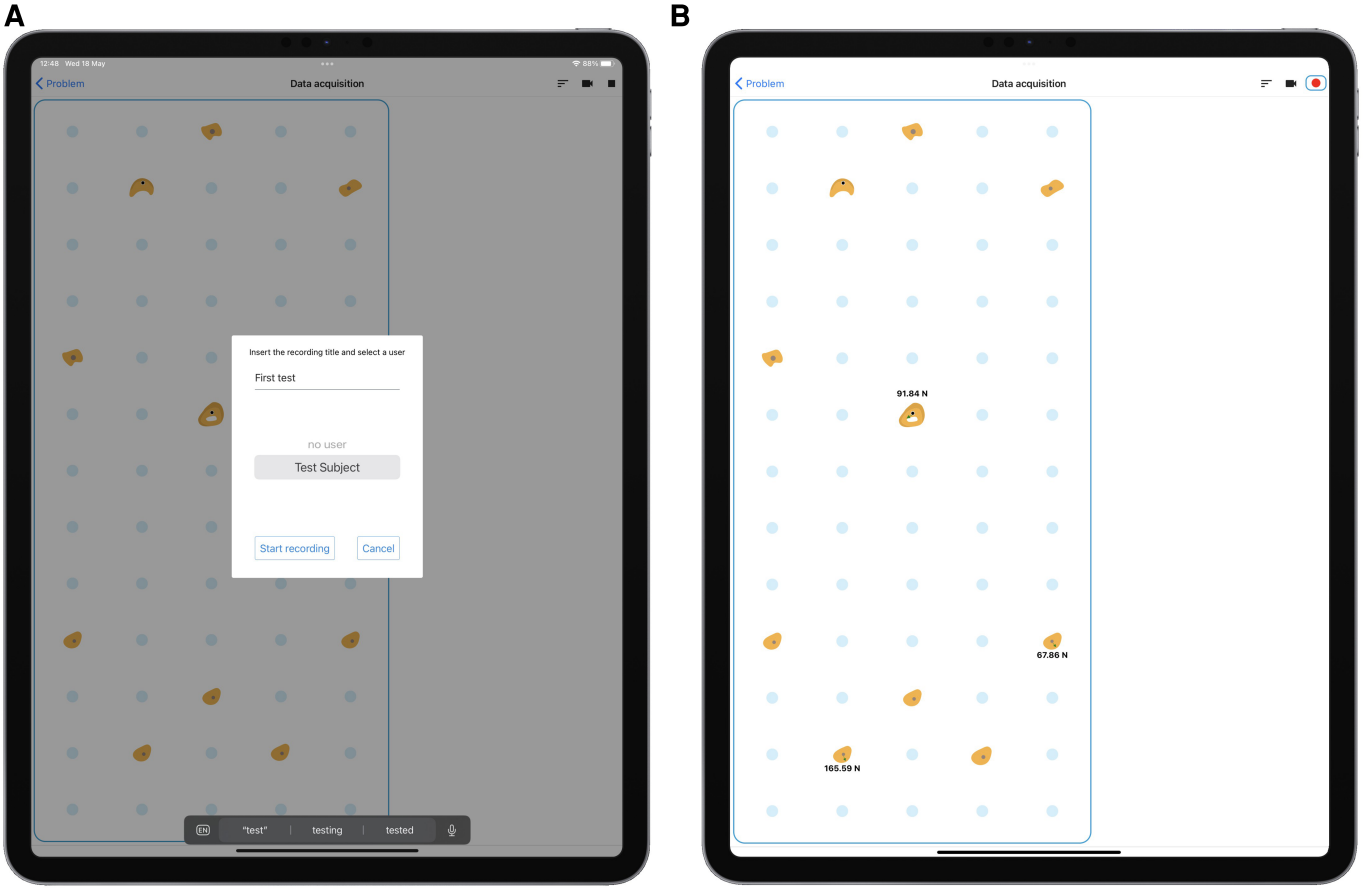
Data Acquisition environment. (**A**) User selection; (**B**) live recording.

The Data Acquisition environment, in Figure 5, is used to handle data recording. It shows a schematic representation of the climbing wall with the holds, positioned as defined through the Setup environment, with an overlay reporting the measured forces in real-time, and it allows to start and stop a recording and assign it a name.

**Figure 6:**
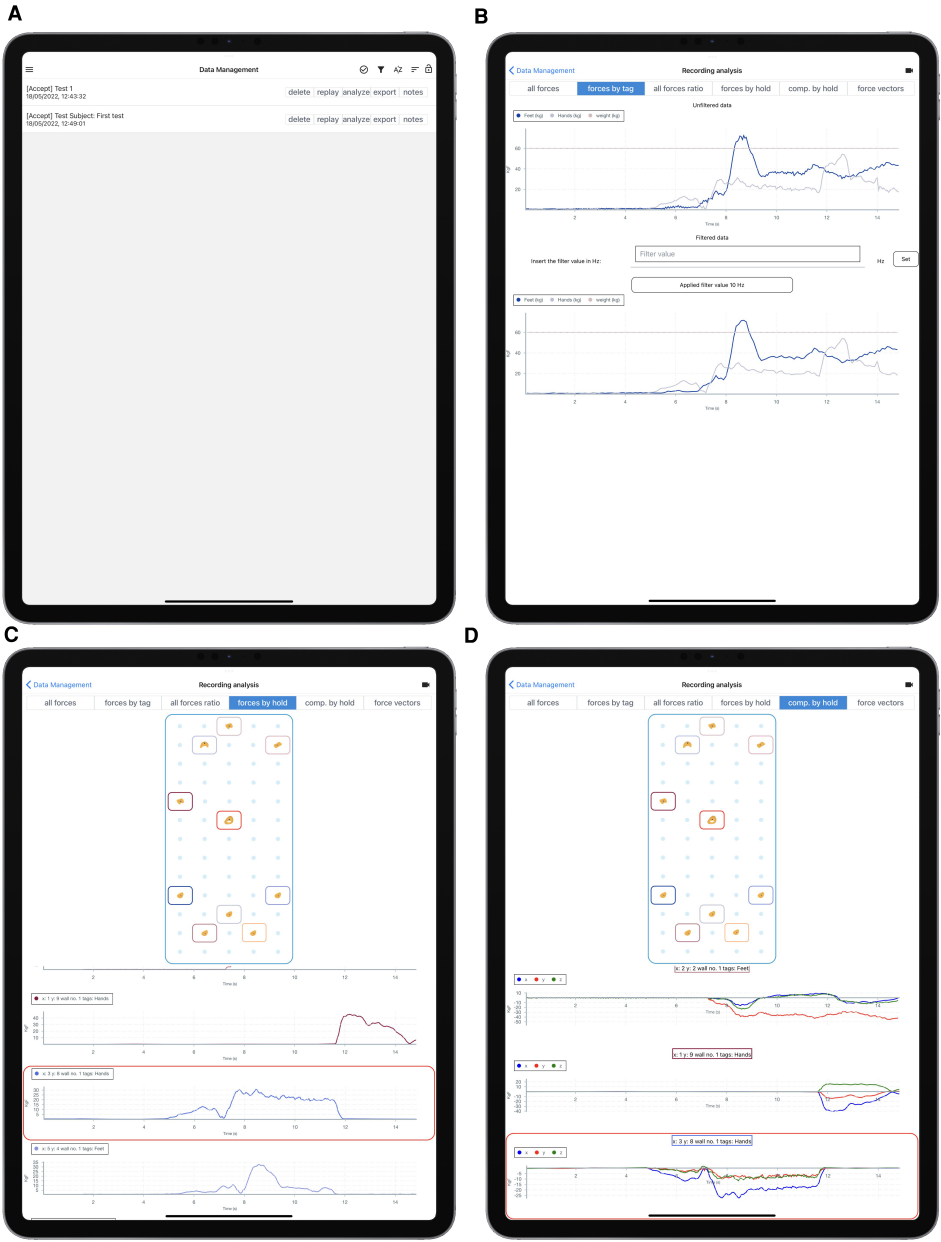
Data Management and Recording Analysis environment. Top to bottom and left to right: record selection, visualization of the magnitude of the vector sum of all forces on holds tagged “hands” or “feet,” compared with climber body weight as inserted in the User Management environment, visualization of the magnitude of the force measured on each hold, visualization of the three components of the force measured on each hold.

The Data Management and Recording Analysis environment, in Figure 6, proposes different views of the recorded data and allows to export it in JSON format. Panel A shows the Data Management menu, where recordings can be selected for analysis, exported, or deleted. The other three panels show three preview modes. Implemented preview modes allow to select an arbitrary lowpass filter bandwidth, to visualize the magnitude of the vector sum of all forces measured on holds with a given tag (in our example: *Feet* and *Hands*, panel B), to visualize the magnitude of the force measured on each hold (panel C), and to visualize the three components of the force measured on each hold (panel D, x in blue, y in red, z in green). In the last two modes, the user can tap on a hold in the visual representation of the grid, to highlight the corresponding set of plots. In the screenshot, the central hold, in position x=3, y=8, has been tapped and is highlighted in red, together with the corresponding plots. Note that, in all these screenshots, forces are expressed in kgf according to the experts’ preference. Data is however recorded in N, and the analyses that we performed on the exported data and report in later sections utilize SI units.

To bring client and server components together, we decided to leverage a local WiFi network where both client and server are connected. We opted for WiFi instead of Bluetooth as this increases the possibilities in terms of deployment of the application and does not require executing native code that needs to be signed and distributed through Apple’s official channels (both for production, development, and testing of the application). Such a local network can, but does not have to, be connected to the internet.

### Participants

In order to test the functionality of the hardware and software setup in a realistic data acquisition session, we recruited a total of 11 adult climbers. The study was approved by the Ethics Committee of Politecnico di Milano, and all participants read and completed an informed consent form. We report in Table 2 their declared years of climbing experience, and maximum redpoint grade reached in the 3 years prior to the experiment, in the French/sport scale (see e.g., (38) for a description climbing grade scales). Climbers 5 and 6, classified as *novice* in the table, were at their first experience on a climbing wall.

**Table 2:** Climbers who participated in the test: years of climbing experience and maximum redpoint grade in the last 3 years.

Climber	Years of climbing experience	Max lead redpoint (French/sport)
1	6	7b
2	7	7a
3	15	8a
4	18	7c
5	novice	-
6	novice	-
7	4	5b
8	4	6a
9	10	6c
10	49	6c
11	11	6a

### Experimental design

We initially characterized the force sensor performances on a bench-top hydraulic tensile/compressive testing rig (MTS 858 Mini Bionix II) to evaluate sensor linearity, hysteresis, and crosstalk, for loads directed along the sensor’s x, y, z axis. A single sensor was tested. Force sensors are required to measure the force vector irrespective of the point of application on the hold, that is, irrespective of eccentricity. To test this, we loaded the sensor in the x and y direction with an offset of 15 and 90 mm along the z axis from the hold attachment point, and in the z direction with an offset of 0 and 80 mm along the y axis (for definition of coordinate system, see Figure 2). The results are representative of the expected performance when using holds of diameter up to roughly 180 and 58 mm thick, assuming the wall thickness to be 20 mm, plus the 2 mm spacer. All tests were performed with nominal loads $F_{N}$ ranging from 0 to 1600 N in 13 steps (0, 320, 640, 960, 1280, 1600, 1280, 960, 640, 320, 0 N), covering the range of loads identified in the requirements.

**Figure 7:**
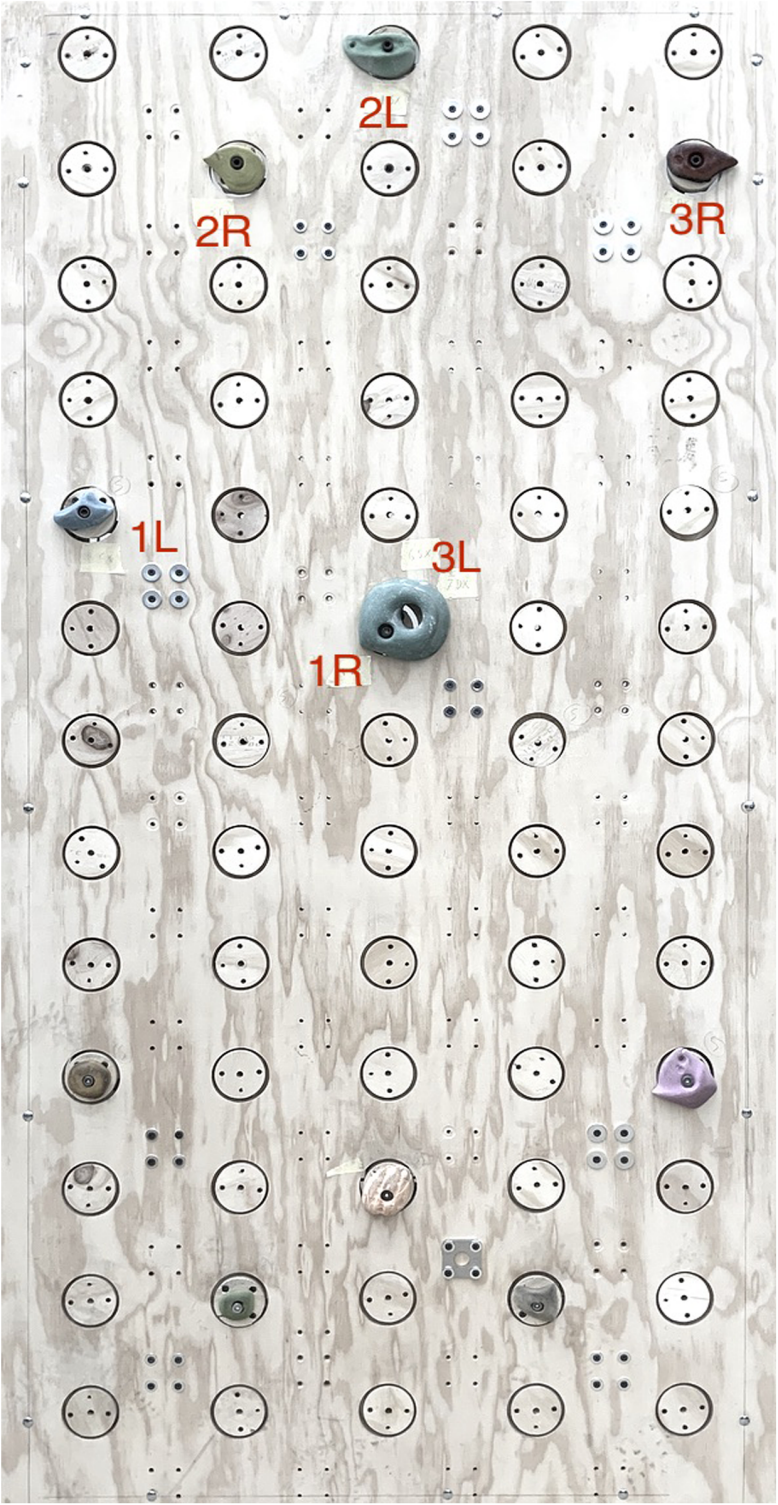
Sensorized wall setup. The labels indicate the sequence of mandatory hand moves that was used in the test, as described in the Sensor network validation Section.

Then, a total of 10 sensors (mechanical component and acquisition board) were assembled and networked (only 10 sensors were available at the time of this testing). The network of sensors was mounted on a wall (see Figure 7) with a front panel adapted to host the 10 sensors in any one of 65 possible locations, arranged in a 5 by 13 grid. The sensorless locations were covered with a wooden lid equipped with standard M10 inserts, though for the purpose of our testing, only the sensorized locations were utilized. Once the sensors were on the wall, the APP setup time for the 10 sensors was less than 10 min.

The sensorized wall was then used as a platform for the climbing exercise, during which data was acquired. All climbers were instructed to perform a short warm-up session on the ground before starting the climbing exercise. The exercise consisted of a circular circuit on the sensorized wall with a fixed sequence of handholds, as depicted in Figure 7: climbers started with right hand on 1R and left hand on 1L and had to move right and left hand alternatively through the sequence 2R-2L-3R-3L-1R-1L, which brought them back to the starting position. Feet placements were not fixed to allow climbers to optimize their moves as they practiced the circuit. The wall angle was set at $90^{\circ }$. Climbers had no prior experience with the circuit before the test and were instructed to repeat the circular circuit as long as they could endure.

## Results

### Force sensor characterization

The results of the characterization of the sensor are reported in Tables 3 and 4.

**Table 3:** Linearity, hysteresis, and eccentricity errors with nominal force $F_{N}$ ranging between 0 and 1600 N. Error bounds expressed as a percent of $F_{N}$.

	$F_{x}\;(\text{\%}F_{N})$	$F_{y}\;(\text{\%}F_{N})$	$F_{z}\;(\text{\%}F_{N})$
Linearity ≤	0.16	0.21	0.14
Hysteresis ≤	0.23	0.17	0.23
Eccentricity ≤	0.40	1.79	0.84

**Table 4:** Crosstalk errors with a nominal force ranging between 0 and 1600 N. Error bounds expressed as a percent of $F_{N}$.

	Applied force direction		
	x	y	z
Crosstalk $F_{x}\;(\text{\%}F_{N})$ ≤		2.20	3.95
Crosstalk $F_{y}\;(\text{\%}F_{N})$ ≤	2.75		1.09
Crosstalk $F_{z}\;(\text{\%}F_{N})$ ≤	3.65	2.26	

One further performance metric regards the noise characteristic of the sensor. As mentioned before, the force sensors sample at 80 Hz. Without further filtering, during lab acquisitions, we measured a Gaussian noise (Kolmogorov Smirnov one-sample test) with a standard deviation less than 1 N. This suggests that the sensors will be able to resolve load signals of amplitude well below 5 N with bandwidth below 40 Hz, which is where we expect to find most of the information generated by human motion.

### Sensor network functional validation

**Figure 8:**
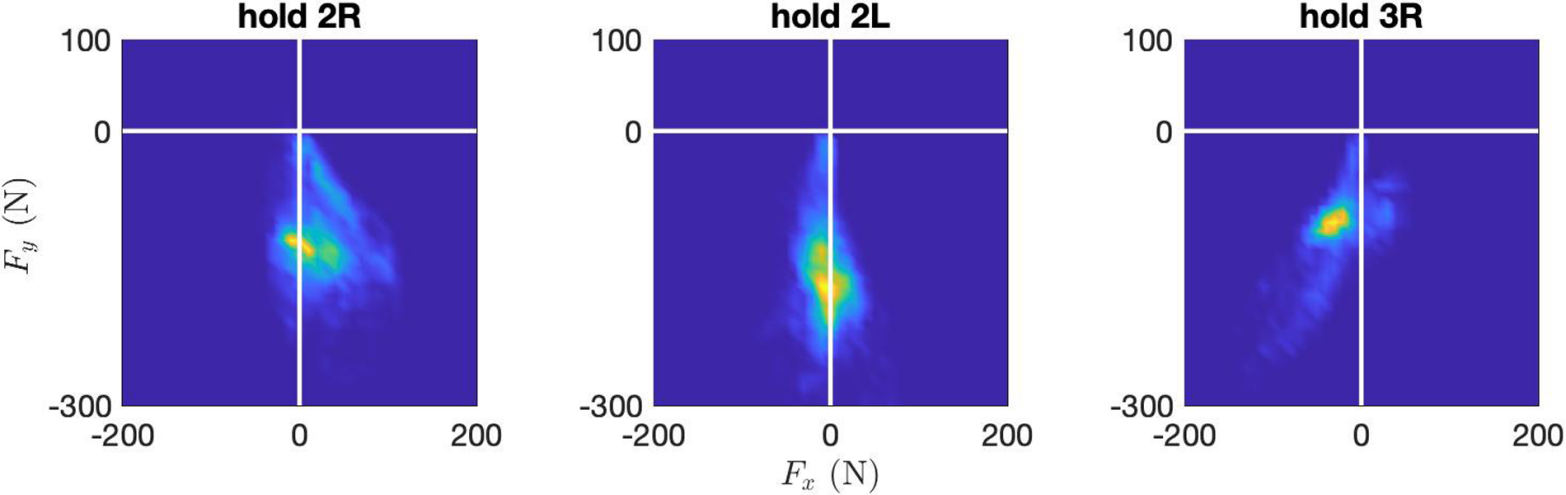
Force density plot on holds 2R, 2L, 3R, during the trial with Climber 4, which lasted about 9 min. Heat map proportional to the total time a given force value was measured on the hold.

**Figure 9:**
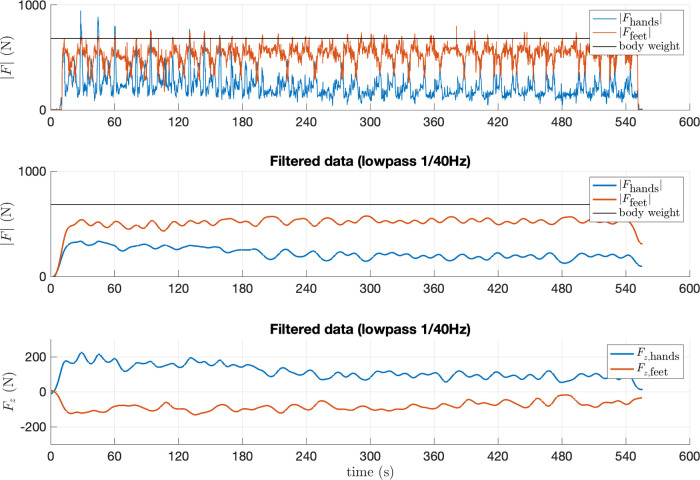
The two top panels display the magnitude of the total force measured on holds tagged *Hands* and *Feet*, during the trial with Climber 4, which lasted about 9 min. In the top panel, the force magnitude is plotted without prior filtering, in the central panel it is low-pass filtered at 1/40 Hz. The bottom panel shows the sum of the $F_{z}$ components (orthogonal to the climbing wall, positive outward) measured on all handholds (blue) and footholds (red), low-pass filtered at 1/40 Hz.

**Figure 10:**
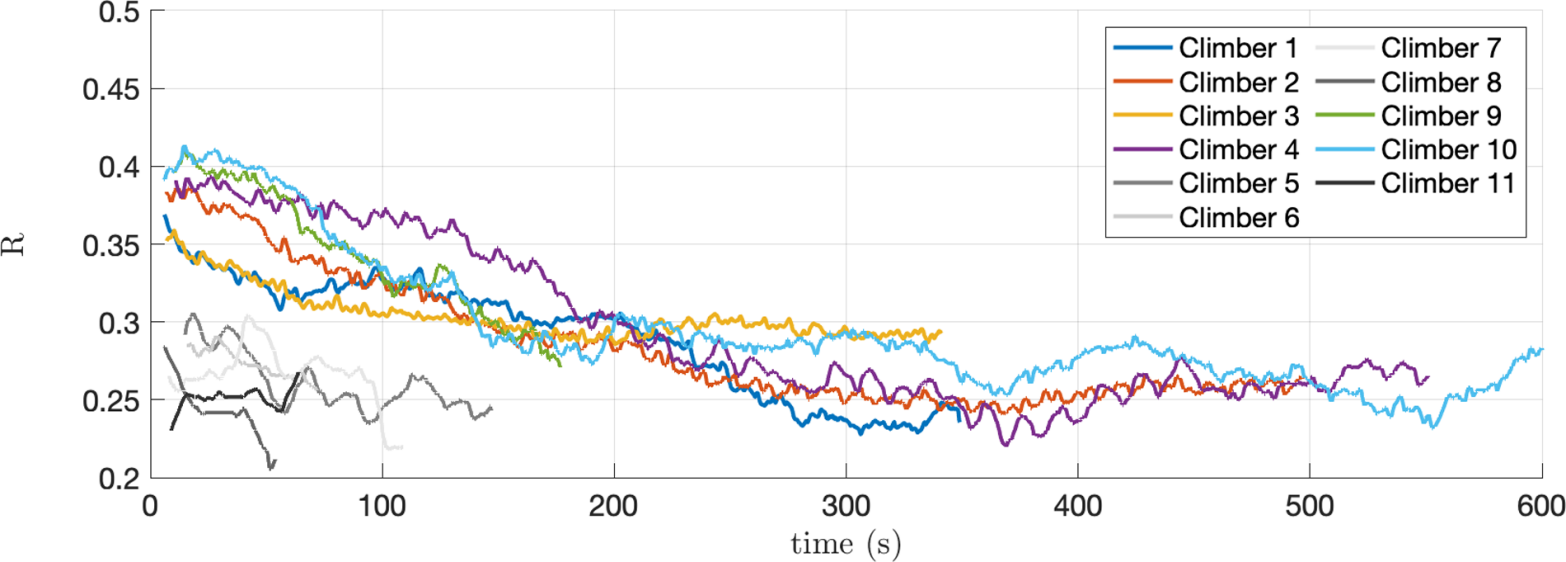
Hands to total force ratio R(t), defined in Eq. (1), for the 11 test climbers.

All climbers reported that the slight movement of the holds due to the sensor deformation under load, and the gap between the hold and the wall, were not perceivable during the climb. The acquisition app collected the simultaneous data streams from the 10 sensors, sampled at 80 Hz, without data loss, as expected. The drift in the time stamps attached to each data point by the acquisition boards with respect to an external clock was not detectable, throughout the length of the experiment. Measures returned by each hold allowed to clearly discriminate the direction and intensity of the force in relation to the task and the position of the hold. See, as an example, Figure 8, relative to the trial with Climber 4, which lasted about 9 min. Here, hold 2L was mostly loaded along the vertical axis, while holds 2R and 3R, which were used to move up and down between 1R/3L and 2L, respectively, show a significant $F_{x}$-component, as one would expect to balance the weight transition between feet. Reference system alignment and prior tagging allowed for simple post-processing of the data. In our test (see Figure 9) this allowed us to compute the magnitudes $\left|F_{\mathrm{hands}}(t)\right|$ and $\left|F_{\mathrm{feet}}(t)\right|$ of the vector sum of the forces on all holds tagged Hands and Feet at time t, to discriminate the contribution of upper and lower limbs. A similar process was followed for Figure 10, which displays the hands to total force ratio R(t) , for the 11 test climbers, defined as

(1) $$R(t)=F_{\mathrm{hands},\mathrm{avg}}(t)/(F_{\mathrm{hands},\mathrm{avg}}(t)+F_{\mathrm{feet},\mathrm{avg}}(t)).$$

In the above formula, $F_{\mathrm{hands},\mathrm{avg}}(t)$ and $F_{\mathrm{hands},\mathrm{avg}}(t)$ are the moving average, over a 60s window, of $\left|F_{\mathrm{hands}}\right|$ and $\left|F_{\mathrm{feet}}\right|$, respectively.

## Discussion

We have illustrated the design process of a complete acquisition system for the analysis of climbing motion in a naturalistic environment. The design needs were collected through semi-structured interviews with 8 experts in different fields, which were then processed into the requirements reported in Table 1.

Through these interviews, we identified force sensors, embedded in the climbing wall, as the most effective option to provide complementary information to that which is typically obtained by trainers or therapists observing a climbing exercise. In order to obtain a robust system at a reasonable cost, we engineered the force sensors from the ground up, leading to a design that is illustrated and validated in the paper, that is highly optimized for the task. The resulting design utilizes a sensing element with simple geometry (to reduce costs) equipped with strain gauges. A dedicated acquisition board is attached to each sensor, which becomes a node in a CAN network. This greatly simplifies the task of moving sensors to different locations on the wall, even though, to reach the level of simplicity that was requested from our interviews, we believe that a wall with fixed (and therefore numerous) sensors would be necessary.

A relevant unknown in our design was related to the impact that the sensor deformation, and the 2mm step between the sensor surface and the wall surface, would have on the climber’s perception. After our tests, climbers reported not noticing any difference with respect to a standard wall. It is however possible that a high-level climber may use the 2mm step between the sensor’s frontal surface and the wall as an additional grip, especially for foot traction.

The interviews also highlighted how a simple user interface was needed, to simplify both the reconfiguration of the climbing exercises and the recording and visualization of the data. To meet the requirements, we designed the acquisition system so that it communicates all recorded data to an App that can be run on a portable device, and we organized the App around 4 fundamental environments: Setup, User Detail, Data Acquisition, Data Management and Recording Analysis. This structure maps the user interface onto the main moments of the interface usage (wall configuration, identification of the athletes, acquisition, and data analysis, respectively), and allows the optimization of the interface for the specific requirements of each of these moments.

To test the system we built a batch of 10 identical sensors, and we characterized one using a hydraulic tensile/compressive testing rig. Linearity and hysteresis were well below 0.3% over the full measuring range. Moreover, measures changed by less than 1% when force is applied with up to 90 mm of offset from the attachment point, and the crosstalk is below 4% between all channels. The performance is slightly inferior to that obtained through other designs (e.g., (21)) but still adequate for most purposes, with the great advantage of having a single-piece sensor body, which simplifies production and reduces costs (for a 3-DOF sensor). Since we characterized a single sensor, it might possible to observe some variability of these parameters within the batch, though the industrial production process should guarantee reasonable repeatability. Quantification of the inter-sensor variability ought to be one of the next steps in the research.

We tested the acquisition system functionality by organizing a realistic data acquisition campaign, involving 11 climbers. The system setup, including the placement of the sensors and the configuration of the wall, proved simple and relatively fast, requiring approximately 10 min to configure the app with 10 sensors and place the holds.

Climber 4, whose exercise was analyzed in detail as an example, tended to load hold 2L heavier than holds 2R and 3R (Figure 8). Hold 3R was typically loaded with a slightly negative $F_{x}$ component (i.e., pulling slightly to the left), while hold 2R was loaded with a slightly positive $F_{x}$ component. These observations, unsurprising given the task, confirm that the resolution of the sensor allows to discriminate the subtle differences in the way the athlete loads a hold (in this case, three similar crimps) when executing different movements. Furthermore, the climber loaded the handholds heavily at the beginning of the exercise (Figure 9, top panel), but the magnitude of the force spikes on handholds tended to decrease as the climber tired. There is, in other words, a visible decreasing trend in the average force on handholds, and an increasing trend of the average force on footholds, which is confirmed in the central panel, which shows the same signals fed through a lowpass filter with bandwidth 1/40 Hz (using Matlab’s *lowpass* function with default parameters). The data shows the climber’s clear tendency to gradually shift load from hands to feet, in a trend that roughly lasts the whole of the first 4 min of the trial. This was probably obtained by slightly changing the pose, and by learning to keep the center of mass closer to the wall. This hypothesis is supported by observing, in the lower panel, the plot of the z components of the vector sum of all forces measured by handholds or footholds. We can see that in the first part of the climb, hands exert an $F_{z}$ of nearly 200 N (positive, i.e., pull out of the wall), which is balanced by an opposite z component on the feet. Throughout the climb, as the climber learns to hold the center of mass closer to the wall, these two components decrease in magnitude.

Interestingly, and quite unexpectedly, this trend was common to the tested higher-level climbers (advanced and elite level according to the IRCRA reporting scale) but was less visible in lower-level ones. Climbers 5, 6, 7, 8, and 11, whose plots are in grayscale in Figure 10, were on the lower end of climbing expertise according to their declared maximum redpoint grade (Table 2, max lead redpoint ≤6a). Their exercises lasted between 48 s (climber 8) and 132 s (climber 5). The remaining, higher-level climbers (Table 2, max lead redpoint ≥6c) remained on the wall between 167 s (climber 9) and 659 s (climber 10), and the plots of their R(t) are reported in color. We can observe that the group of higher-level climbers started the exercise with a higher R(t), which tends to decrease in time, appearing to settle within a similar range as that of the lower-level climbers. In other words, higher-level climbers appear to use upper-limb force relatively more than lower-limb force at the beginning of the exercise, contrary to the common belief that more experienced climbers are better at optimizing weight distribution.

A quantitative assessment of this trend is reported in Table 5. Data for the group of lower-level climbers is reported but grayed out since the duration of their exercise is very short and, consequently, the confidence intervals are relatively large. Among the higher-level climbers, climbers 2, 3, 4, and 10 have asymptotes (coefficient a) located between 0.24 and 0.29, with time constants (−1/c) ranging between 56 s (1/0.018) and 188 s (1/0.0055). Their R(t), therefore, settles in a few minutes to a range that is close to that expressed from the outset by the group of less experienced climbers. This may indicate an effect of learning or fatigue. Climbers 1 and 9 instead appear to follow a very different pattern, though they still exhibit a decreasing trend. Even though the primary objective of this study was to verify the feasibility of a cost-effective measurement system for sport climbing that is suitable to be used in a naturalistic setting (i.e., in a climbing gym), and not to test a specific performance index, the data that was collected during the tests, therefore, revealed some unexpected features, which may be worthy of further investigation. A systematic study, involving a larger sample size would be needed to properly address this issue. More directly relevant to the context of this paper, this observation was linked to a gradual reduction of the z component (orthogonal to the climbing wall) of the total force, indicating that climbers were gradually moving their center of mass closer to the wall. This supports our choice of reducing costs by simplifying the design of- 3DOF sensors, rather than by recurring to 1 or 2-DOF sensors as suggested in (22), which would not be able to discern forces in the z direction.

**Table 5:** Coefficients of a nonlinear least square fit of the function $a+be^{ct}$ to the curves in Figure 10, 95% confidence interval bounds of the coefficients, and the duration of the exercise.${}^{a}$

Climber	a (95% C.I.)	b (95% C.I.)	c (95% C.I.)	Duration (s)
1	−0.64 (−0.86,−0.43)	1 (0.79,1.2)	−0.00038 (−0.0046,−0.0029)	343
2	0.25 (0.25,0.25)	0.16 (0.16,0.16)	−0.0078 (−0.0078,−0.0078)	490
3	0.29 (0.29,0.29)	0.073 (0.072,0.073)	−0.018 (−0.018,−0.018)	334
4	0.24 (0.24,0.24)	0.20 (0.19,0.20)	−0.0055 (−0.0056,−0.0055)	540
5	0.25 (0.25,0.25)	0.11 (0.10,0.11)	−0.033 (−0.034,−0.032)	132
6	−0.46 (−2.2,1.3)	0.76 (−0.97,2.5)	−0.00070 (−0.0023,0.00094)	56
7	−0.72 (−13,12)	1.0 (−11,13)	−0.00028 (−0.0038,0.0033)	101
8	0.19 (0.18,0.19)	0.099 (0.093,0.10)	−0.020 (−0.023,−0.018)	48
9	−0.43 (−0.54,−0.33)	0.85 (−0.75,0.96)	−0.0010 (−0.0012,−0.00089)	167
10	0.27 (0.27,0.27)	0.18 (0.18,0.18)	−0.0091 (−0.0091,−0.0090)	659
11	−0.0092 (−6.7,6.7)	0.26 (−6.5,7)	0.00045 (−0.011,0.012)	55

${}^{a}$Provided that c is negative, a represents the asymptotic value of the interpolating function, while −1/c represents the time constant, i.e., it is proportional to the time needed for the exponential interpolating function to approximately reach its asymptotic value.

Overall, the design illustrated in this paper meets the requirements that were identified during our interviews. The resulting system is essentially transparent to the climber and relatively simple to use. The limited cost of the sensors makes it possible to sensorize a large number of placements at a reasonable cost, thus reducing the need to physically move the sensors, which proved to be the most time-consuming task when reconfiguring the sensorized wall. The arrangement of the sensors in a CAN bus network and the logical structure of the App simplify the handling of multiple climbing problems defined over subsets of the sensors network. A single CAN network is usually expected to work flawlessly with up to about 40 different nodes or more, but the server architecture on which our system is based can easily be extended to handle multiple CAN buses. Thus, technically, extending such an architecture to a few hundred sensorized holds should be possible. A more relevant limitation is in the size of the holds that can be mounted on the sensors. Modern sport climbing is frequently using large volumes, and speed climbing uses holds that are much bigger than those for which our sensors were designed and tested. These limitations notwithstanding, a public wall equipped with a grid of sensors could provide climbing instructors and therapists with a new and very valuable tool to complement their observation, and help develop a more quantitative understanding of the subtleties of climbing motion.

## Data Availability

The raw data supporting the conclusions of this article will be made available by the authors, without undue reservation.

## References

[1] Cook E. Percentage baseball. Massachussets (USA): MIT Press (1964).

[2] Lightman K. Silicon gets sporty. IEEE Spectr. (2016) 53:48–53. 10.1109/MSPEC.2016.7420400

[3] Sharma A Al-Dala’in T Alsadoon G Alwan A. Use of wearable technologies for analysis of activity recognition for sports. *2020 5th International Conference on Innovative Technologies in Intelligent Systems, Industrial Applications (CITISIA)*. IEEE (2020).

[4] Hadzic V Germic A Filipcic A. Validity, reliability of a novel monitoring sensor for the quantification of the hitting load in tennis. PLoS ONE. (2021) 16:1–13. 10.1371/journal.pone.0255339 PMC8321100 34324580

[5] Kos A Wei Y Tomažič S Umek A. The role of science, technology in sport. Procedia Comput Sci. (2018) 129:489–95. 2017 International Conference on Identification, Information, Knowledge in the Internet of Things. 10.1016/j.procs.2018.03.029

[6] Kos A Umek A. Smart sport equipment: smartski prototype for biofeedback applications in skiing. Pers Ubiquitous Comput. (2018) 22:535–44. 10.1007/s00779-018-1146-1

[7] Watts PB. Physiology of difficult rock climbing. Eur J Appl Physiol. (2004) 91:361–72. 10.1007/s00421-003-1036-7 14985990

[8] Quaine F Martin L Blanchi J. Effect of a leg movement on the organisation of the forces at the holds in a climbing position 3-D kinetic analysis. Hum Mov Sci. (1997) 16:337–46. 10.1016/S0167-9457(96)00060-7

[9] Quaine F Martin L Blanchi JP. The effect of body position and number of supports on wall reaction forces in rock climbing. J Appl Biomech. (1997) 13:14–23. 10.1123/jab.13.1.14

[10] Quaine F Martin L. A biomechanical study of equilibrium in sport rock climbing. Gait Posture. (1999) 10:233–9. 10.1016/S0966-6362(99)00024-7 10567755

[11] Noé F Quaine F Martin L: Influence of steep gradient supporting walls in rock climbing: biomechanical analysis. Gait Posture. 13 (2001) 86–94. 10.1016/S0966-6362(00)00098-9 11240356

[12] Fuss FK Niegl G. Biomechanics of the two-handed dyno technique for sport climbing. Sports Eng. (2010) 13:19–30. 10.1007/s12283-010-0052-1

[13] Boulanger J Seifert L Herault R Coeurjolly JF. Automatic sensor-based detection and classification of climbing activities. IEEE Sens J. (2016) 16:742–9. 10.1109/JSEN.2015.2481511

[14] Quaine F Vigouroux L Martin L. Effect of simulated rock climbing finger postures on force sharing among the fingers. Clin Biomech. (2003) 18:385–8. 10.1016/S0268-0033(03)00045-7 12763433

[15] Amca AM Vigouroux L Aritan S Berton E. Effect of hold depth and grip technique on maximal finger forces in rock climbing. J Sports Sci. (2012) 30:669–77. 10.1080/02640414.2012.658845 22339482

[16] Vigouroux L Devise M Cartier T Aubert C Berton E. Performing pull-ups with small climbing holds influences grip and biomechanical arm action. J Sports Sci. (2018) 37:886–94. 10.1080/02640414.2018.1532546 30326778

[17] van Bergen NG Soekarjo K Van der Kamp J Orth D. Reliability and validity of functional grip strength measures across holds and body positions in climbers: associations with skill and climbing performance. Res Q Exerc Sport. (2022):1–11. 10.1080/02701367.2022.2035662 35452375 PMC10503502

[18] Stien N Vereide VA Saeterbakken AH Hermans E Shaw MP Andersen V. Upper body rate of force development, maximal strength discriminates performance levels in sport climbing. PLoS ONE. (2021) 16:1–13. 10.1371/journal.pone.0249353 PMC7997018 33770128

[19] Fuss FK Niegl G. The fully instrumented climbing wall: performance analysis, route grading, vector diagrams: a preliminary study. *The impact of technology on sport II*. CRC Press (2007). p. 677–682.

[20] Fuss FK Niegl G. Instrumented climbing holds and performance analysis in sport climbing. Sports Technol. (2008) 1:301–13. 10.1080/19346182.2008.9648487

[21] Bauer F Simnacher M Stöcker U Riener R Wolf P. Interaction forces in climbing: cost-efficient complementation of a 6dof instrumentation. Sports Technol. (2014) 7:120–7. 10.1080/19346182.2015.1064127

[22] Baláš J Panáčková M Jandová S Martin AJ Strejcová B Vomáčko L, et al. The effect of climbing ability and slope inclination on vertical foot loading using a novel force sensor instrumentation system. J Hum Kinet. (2014) 44:75–81. 10.2478/hukin-2014-0112 25713667 PMC4327382

[23] Donath L Wolf P. Reliability of force application to instrumented climbing holds in elite climbers. J Appl Biomech. (2015) 31:377–82. 10.1123/jab.2015-0019 25950657

[24] Pandurevic D Sutor A Hochradel K. Methods for quantitative evaluation of force and technique in competitive sport climbing. J Phys Conf Ser. (2019) 1379:012014. 10.1088/1742-6596/1379/1/012014

[25] Maffiodo D Sesana R Gabetti S Colombo A. Innovative force sensor for indoor climbing holds – real-time measurements and data processing, design and validation. Proc Inst Mech Eng P. (2020) 234(4):298–311. 10.1177/1754337120927122

[26] Stien N Saeterbakken AH Hermans E Vereide VA Olsen E Andersen V. Comparison of climbing-specific strength and endurance between lead and boulder climbers. PLoS ONE. (2019) 14:1–13. 10.1371/journal.pone.0222529 PMC6752829 31536569

[27] Reveret L Chapelle S Quaine F Legreneur P. 3D visualization of body motion in speed climbing. Front Psychol. (2020) 11. 10.3389/fpsyg.2020.02188 33117209 PMC7549505

[28] Wright DM Royle TJ Marshall T. Indoor rock climbing: who gets injured? Br J Sports Med. (2001) 35:181–5. 10.1136/bjsm.35.3.181 11375878 PMC1724320

[29] Lutter C Hotfiel T Tischer T Lenz R Schöffl V. Evaluation of rock climbing related injuries in older athletes. Wilderness Environ Med. (2019) 30:362–8. 10.1016/j.wem.2019.06.008 31668938

[30] Jones G Asghar A Llewellyn DJ. The epidemiology of rock-climbing injuries. Br J Sports Med. (2008) 42:773–8. 10.1136/bjsm.2007.037978 18065444

[31] Schuler D Namioka A. Participatory design: principles and practices. Boca Raton, Florida (USA): CRC Press (1993).

[32] Sanders EBN Stappers PJ. Co-creation and the new landscapes of design. CoDesign. (2008) 4:5–18. 10.1080/15710880701875068

[33] Steen M. Tensions in human-centred design. CoDesign. (2011) 7:45–60. 10.1080/15710882.2011.563314

[34] Mencarini E Leonardi C Cappelletti A Giovanelli D Angeli AD Zancanaro M. Co-designing wearable devices for sports: the case study of sport climbing. Int J Hum Comput Stud. (2019) 124:26–43. 10.1016/j.ijhcs.2018.10.005

[35] Black IS Fennelly L. Interviewing techniques. *Investigations and the art of the interview*. Oxford (UK) or Waltham, Massachussets (USA): Butterworth-Heinemann (2021). Chap. 10.

[36] Bogner A Littig B Menz W. Interviewing experts. London (UK): Palgrave Macmillan (2009).

[37] [Dataset] En 12572-2:2017: Artificial climbing structures - part 2: Safety requirements and test methods for bouldering walls (2017).

[38] Draper N Giles D Schöffl V Fuss FK Watts P Wolf P, et al. Comparative grading scales, statistical analyses, climber descriptors and ability grouping: international rock climbing research association position statement. Sports Technol. (2015) 8:88–94. 10.1080/19346182.2015.1107081

